# Variation in Seed Dormancy Quantitative Trait Loci in *Arabidopsis thaliana* Originating from One Site

**DOI:** 10.1371/journal.pone.0020886

**Published:** 2011-06-30

**Authors:** Rebecca A. Silady, Sigi Effgen, Maarten Koornneef, Matthieu Reymond

**Affiliations:** 1 Max Planck Institute for Plant Breeding Research, Cologne, Germany; 2 Laboratory of Genetics, Wageningen University, Wageningen, The Netherlands; University of California, United States of America

## Abstract

A Quantitative Trait Locus (QTL) analysis was performed using two novel Recombinant Inbred Line (RIL) populations, derived from the progeny between two *Arabidopsis thaliana* genotypes collected at the same site in Kyoto (Japan) crossed with the reference laboratory strain Landsberg *erecta* (L*er*). We used these two RIL populations to determine the genetic basis of seed dormancy and flowering time, which are assumed to be the main traits controlling life history variation in Arabidopsis. The analysis revealed quantitative variation for seed dormancy that is associated with allelic variation at the seed dormancy QTL *DOG1* (for *Delay Of Germination 1*) in one population and at *DOG6* in both. These *DOG* QTL have been previously identified using mapping populations derived from accessions collected at different sites around the world. Genetic variation within a population may enhance its ability to respond accurately to variation within and between seasons. In contrast, variation for flowering time, which also segregated within each mapping population, is mainly governed by the same QTL.

## Introduction

Seed dormancy is an important adaptive trait that together with flowering time is a primary component of the different life history strategies of plants. *Arabidopsis thaliana* is a species which mainly occupies disturbed areas where there is little competition and completes its life cycle within a relatively short period of time. In moderate climates, such as those in western and central Europe or Japan, *Arabidopsis thaliana* grows during the first half of the year. This necessitates that after seed dispersal in early summer, seeds should not immediately germinate, even when conditions for germination, such as enough moisture, are favourable. Rather they should remain dormant until the fall. Seed dormancy is a mechanism that allows for this survival of seeds and because this trait is under selection [1], insight into the pattern of variation in nature is of interest. A number of studies have identified Quantitative Trait Loci (QTL) for seed dormancy in Arabidopsis. These QTL studies were performed using bi-parental mapping populations derived from single homozygous individuals collected from various parts of the world [2]. QTL analysis revealed that when moderately or strongly dormant accessions are compared with the relatively low-dormant laboratory strain Landsberg *erecta* (L*er*), two loci are mainly involved, named *Delay Of Germination (DOG)* 1 and 6 located on chromosome 5 and 3 respectively [2]. Flowering time variation has been studied in the progeny of many crosses as well and overall more than 15 QTL have been revealed of which at least 10 have been identified molecularly [3]. Many of the very late flowering accessions carry functional alleles of *FRIGIDA* for which early flowering genotypes often have loss of function alleles [4].

Arabidopsis is predominantly self pollinated, which implies that natural populations are often mixtures of genetically homozygous lines [5], [6]. However, seed material stored in the stock centres is often only comprised of a single or a limited number of such lines. When seeds from several plants of a genetically heterogeneous population are collected, several homozygous lines can co-exist in a single sample. We experienced such a situation with material collected in 1997, from several plants growing at one site at the campus of Kyoto University in Japan by Dr Goto (Sendai, Japan) and provided by him after multiplication of 5 plants from of the sample collected (N. Goto, pers. commun.). Quantitative genetic analyses performed on such material provide insights into the genetic variation present within a local population. This report describes the genetic analysis of two new recombinant inbred line (RIL) populations derived from two different genotypes collected in the same location (Kyoto-Japan) and demonstrates that variation for important life cycle genes may be present in a single collection site.

## Results

**Figure 1:**
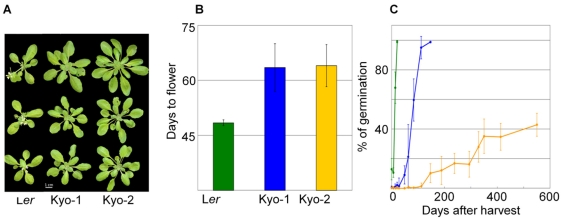
Rosettes of the parental lines (A) and data on flowering time (B) and seed dormancy (C) of the parents.

**Figure 2:**
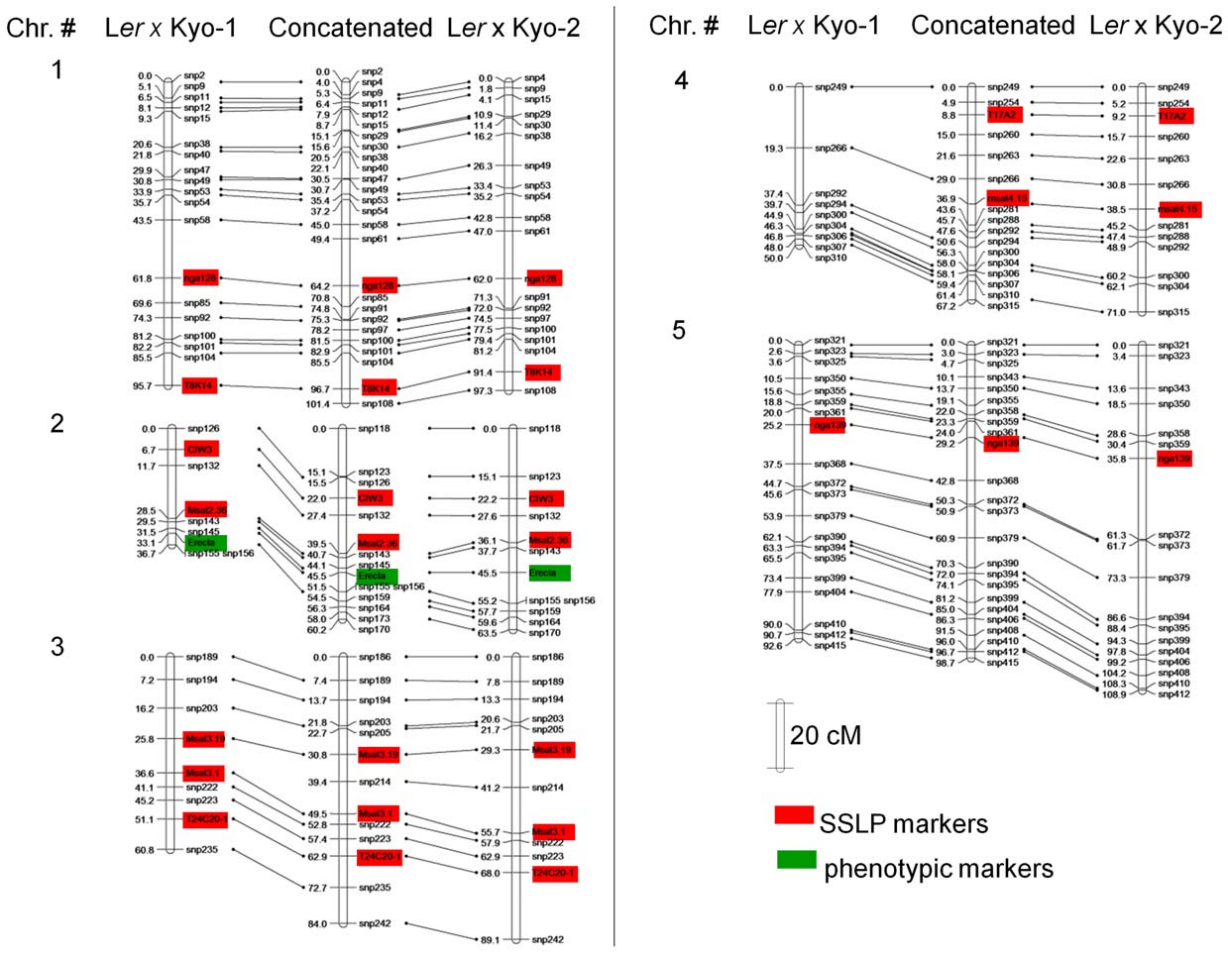
Genetic map of the Ler×Kyo-1 and Ler×Kyo-2 RIL populations and the integrated map derived from both populations.

Several plants from the original JW-137 seed batch were grown in the greenhouse and crossed with the accession Landsberg *erecta* (L*er*). Progenies were developed from single F1 plants. The Kyoto plants showed only minor morphological variation, in the range of what is commonly observed for an inbred line grown in our greenhouse conditions. They were therefore expected to represent a single homozygous line due to their similar overall phenotype and flowering time (Figure 1A and Fig. 1B). However, when seed dormancy was measured in the selfed progeny of the plant lines that were used for the crosses, distinct phenotypes were observed (Fig. 1C). Kyo-2 seeds stored for 49 weeks and stratified for 5 days had a 95% germinated rate indicating that low germination levels of Kyo-2 without stratification are not due to loss of viability, but rather due to high seed dormancy (data not shown). The two L*er*/Kyo mapping populations each from a single F1 plant appeared to be derived from crosses with the two genotypes, henceforth described as Kyo-1 and Kyo-2. These genotypes were genotyped together with the parental lines (see materials and methods). Genotyping of these plants shed light on the genetic differences between Kyo-1 and Kyo-2 (Table S1). Polymorphisms between L*er* and both Kyo-1 and Kyo-2 allowed us to build genetic maps for both derived populations (Figure 2). The maps of chromosome 2, 3 and 4 are somewhat shorter for the Ler/Kyo-1 population than for both the Ler/Kyo-2 population and other previously described populations [7], [8], [9]. Chromosomal regions with segregation distortion (Figure S1) were noted in the L*er*/Kyo-1 population at the top of chromosome 1 (at markers snp2, snp4 and snp9), the middle of chromosome 2 (markers CIW3, Msat2-36 and snp145) and at the top (markers snp321, snp323 and snp325) and middle (markers snp390, snp394 and snp395) of chromosome 5. In addition, distortion of allelic segregation has also been found at punctual marker positions on the Ler/Kyo-2 map (snp108 on the bottom of chromosome 1 and snp118 on the top of chromosome 2). This distortion is most likely due to miscalling of the allelic information at these markers indicated by the high level of heterozygosity for these markers overall in the L*er*/Kyo-2 lines, which assumed miscalls were therefore not included in the map construction. A consensus map was developed (Figure 2) and used for QTL detection in order to facilitate observation of co-localization between QTL from the two populations.

**Figure 3:**
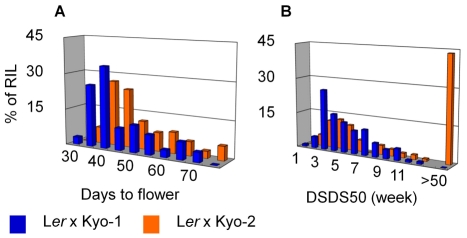
Frequency distributions of trait values for days to flower (A) and DSDS50 (B).

**Figure 4:**
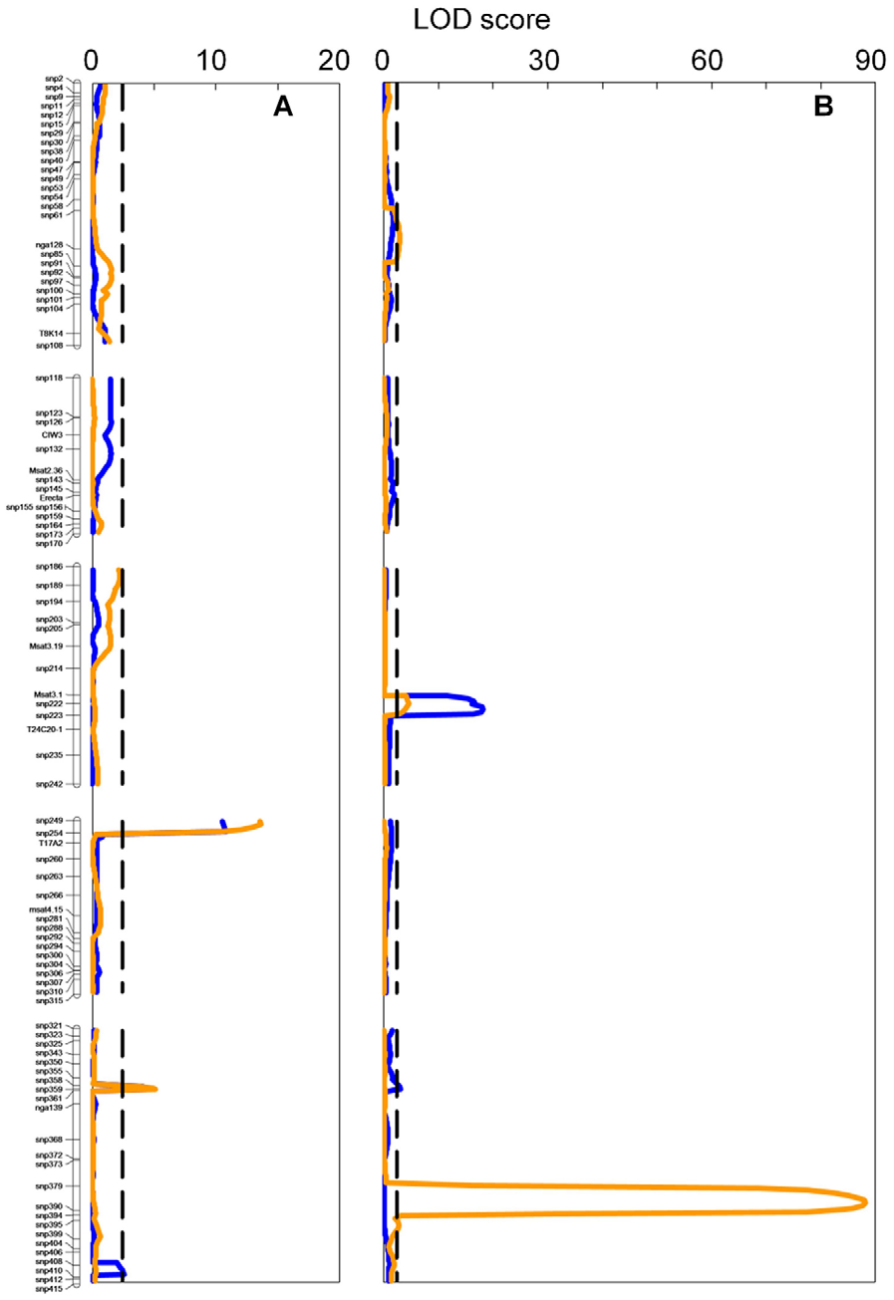
LOD profiles for flowering time (A) and DSDS50 (B) in the Ler×Kyo-1 (in blue) and Ler×Kyo-2 (orange).

QTL analysis was performed for both flowering time and seed dormancy. Flowering time showed ample variation among RILs in both populations (from 20 to 75 days after sowing; Figure 3A) with high heritability (around 0.9 for both populations). Flowering time QTL were mapped (Figure 4A) at the top of chromosome 4 (in both RIL populations) and at two positions on chromosome 5 that were different between the two populations. Variation for seed dormancy was also observed in the mapping populations. In the Kyo-1 population this variation was restricted from 7 to 84 days after harvesting, whereas this variation was spread from 7 days to more than one year in the Kyo-2 population (Figure 3B). Heritabilities for these populations ranged from 0.77 to 0.87. QTL analysis for seed dormancy (Figure 4B) revealed different major QTL in the two populations at positions which have been previously found to harbor seed dormancy QTL in other Arabidopsis mapping populations (named DOG for Delay Of Germination). In the Kyo-1 population the major QTL, *DOG6*, mapped to chromosome 3. The major QTL in the Kyo-2 population, *DOG1*, mapped to the lower part of chromosome 5. *DOG6* was also identified in the Kyo-2 population, though only as a minor QTL (Fig. 4 and Table 1 with QTL effects).

**Table 1:** Characteristics of the QTL for flowering time and seed dormancy detected in the two Ler/Kyo RIL populations.

Trait	QTL	Population	Position			LOD	r^2^ (%)	QTL effect
			Chrom.	cM	Marker			(days)
Flowering time	FT-1	Ler/Kyo-1	4	4.0	snp249	10.8	33.1	−12.9
	FT-1	Ler/Kyo-2	4	1.0	snp249	13.7	44.0	−14.9
	FT-5a	Ler/Kyo-1	5	23.3	snp359	4.4	10.5	−6.9
	FT-5a	Ler/Kyo-2	5	23.3	snp359	5.1	12.8	−8.0
	FT-5b	Ler/Kyo-1	5	96.0	snp410	2.6	5.9	5.2
Delay of Germination	DSDS50-1	Ler/Kyo-2	1	61.4	nga128	2.9	0.1	−9.3
	DSDS50-3	Ler/Kyo-1	3	54.8	snp222	18.2	54.9	−25.3
	DSDS50-3	Ler/Kyo-2	3	52.8	snp222	4.6	0.1	−11.4
	DSDS50-5a	Ler/Kyo-1	5	23.0	snp358	3.1	6.7	8.8
	DSDS50-5b	Ler/Kyo-2	5	67.9	snp390	88.2	94.5	−315.3

## Discussion

Two new RIL populations have been added to the materials that can be used for genetic analysis in Arabidopsis. These were used to map flowering time and seed dormancy, both important life history traits.

The major flowering time QTL on chromosome 4 mapped at similar positions in the 2 mapping populations and co-locates with *FRIGIDA (FRI)*, a QTL previously detected in other *Arabidopsis thaliana* mapping populations. There are two candidate genes for the QTL in the middle of chromosome 5, *HUA2* and *FRL-1*, in both of which, the L*er* parent has a non-functional mutation. At the bottom of chromosome 5 a Kyo-1 specific QTL was detected within the region of the *MAF2-5* cluster of genes [10]. The Kyo-1 allele results in early flowering as found for several Asian accessions [7], [11]. These data show that in these Kyoto accessions no novel QTL for flowering time were found in comparison with populations described before [3] and that variation for flowering time within this population is likely due to genes with minor effects, in addition to the major effect QTL at *FRI*.

The seed dormancy QTL also mapped to loci already shown to be involved in variation of seed dormancy using other mapping populations in *Arabidopsis thaliana* [2]. The QTL on chromosome 3 was detected in both Kyo RIL populations and its confidence interval encompasses *DOG6* found to be a major QTL in RILs derived from the crosses L*er*×Cvi, L*er*×Fei-0, L*er*×Sha, L*er*×Kond and L*er*×Kas-2 [2]. The major QTL detected on the bottom of chromosome 5 in the Kyo-2 population but not in the Kyo-1 population, most likely coincides with the position of *DOG1*, a QTL present in all progenies with strong dormancy [2], [12]. This agrees with the stronger dormancy in the Kyo-2 parent as compared with the Kyo-1 parent. In summary the most striking and large effect genetic differences between the Kyo lines is due to polymorphisms in the major seed dormancy QTL *DOG1*.

The presence of diverse genetic architecture within a population may allow that population to react to variations in environmental conditions. The importance of the timing of seed germination for fitness in nature has been described before [1], [13], [14]. Although the details of the specific *DOG* genotypic and environmental interactions are not yet known, it was recently shown [13] that for a RIL population tested both in field conditions and under similar laboratory conditions as used in this study QTL at the position of both *DOG1* and *DOG6* were identified. In addition, the optimal genotype at these loci was essential for fitness in the field conditions that were tested [1], [13]. The presence of allelic variation at the two major seed dormancy loci in two individuals collected from the same site suggests that when requirements to germinate at the right time may vary within and between seasons, these differences in the genetic architecture provide a system of balancing selection between genotypes for seed dormancy loci. However due to the absence of data on the history and stability of the Kyoto population it cannot be firmly concluded that this observed variation was important to establish and maintain the present population at this collection site.

## Materials and Methods

Seeds from the Kyoto accession JW137 were collected and obtained from Prof N. Goto of the former Sendai resource center now included in BRC (http://www.brc.riken.go.jp/lab/epd/Eng/catalog/seed). Marker analysis (Dr S Iuchi and Dr Kobayashi pers comm.) confirmed that accession JW137 and material collected at two other sites at the campus of Kyoto University (JW138 and JW139) represented 3 distinct genotypes for the BRC collection, with two present in JW137.

Genotyping of the material confirmed that the genotypes used to make the crosses with L*er* had been derived from two different genotypes each represented in one of the two F2 populations. The parental genotypes were also present as pure lines in seeds derived from lines obtained from the original seed batch. Based on SNP marker analysis CS 3964 is identical to Kyo-1 (supplemental Table 1). All lines will be made available through the Arabidopsis stock centres.

Recombinant inbred lines were derived by single seed descent from after selfing the plants of the F2 populations derived from crosses between L*er* (NW20) and plants from the progeny of JW137. Two F8 plants of each recombinant inbred line were grown in an Elbanton growth chamber with temperature set 22°C for 16 hours in the light and 16°C for 8 hours in the dark and use for genotyping with a set of 149 single nucleotide polymorphism (SNP) markers described in [15] by Sequenom, inc. (San Diego, CA). Out of the 149 SNP markers, 83 had good quality data and were polymorphic in one or both of the two populations. In addition 11 SSLP (microsatellite) markers were added and the morphological marker ‘*erecta*’ was scored and included in the genetic map.

The day that the first flower on each plant had visible petals was recorded as the day of flowering. Parental lines were also tested in a separate experiment using similar growth conditions.

Each plant was harvested separately when more than half of the seeds on that particular plant were mature, or when enough mature seeds were present on the plant to perform a seed dormancy assay. Because the population segregated strongly for flowering time, this required that seed harvesting was performed every few days for several weeks. Thereafter seeds were stored at room temperature. Germination tests were performed as described in ref [16] by incubating seeds in 6 cm Petri-dishes on filter paper with sterilized Milli-Q water in a germination incubator (van den Berg, Monfoort, The Netherlands). The germination percentage was determined after imbibing the seeds at 25°C during the 12 hour light period and 20°C during the dark period for 7 days.

Germination was tested for each of the two plants per RIL once a week from the harvest date until nearly 100% of the seeds germinated, except when more than a year passed before the seeds germinated at 100%. The time of dry storage until 50% of the seeds germinated (DSDS50) was estimated by using curve fitting of probit transformed germination percentages as described by Alonso-Blanco et al. [16]. For lines that did not germinate within a year the DSDS50 was set at 350.

Heritability (h^2^) was estimated for each trait by using the following equation:
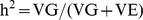
where VG is the variance between PILs and VE is the variance within RIL.

QTL analysis has been performed on average phenotype per line and by using MapQTL5 (Van Ooijen, J.W., 2004 MapQTL®5, Software for the mapping of quantitative trait loci in experimental populations. Kyazma B.V., Wageningen, Netherlands) using the MQM procedure. A permutation test using 1000 permutations of the original data resulted in a genome wide 95% LOD threshold of ∼2.6 for both quantified trait. Markers, used as cofactors, were chosen by backward selection. For each QTL detected, a marker near the QTL was used as a cofactor in the final model (Table 1).

## Supporting Information

Figure S1 Segregation distortion represented by the frequency of each allele in the two mapping populations. * indicates where the deviation from 50% is significant.
(TIF)

Table S1 Marker phenotypes of the parental lines.
(TIF)
