# Supplementary figures and images for: Variation in Seed Dormancy Quantitative Trait Loci in Arabidopsis thaliana Originating from One Site
Source: PLoS One. 2011 Jun 30;6(6):e20886. doi: 10.1371/journal.pone.0020886 (PMC3127951; doi:10.1371/journal.pone.0020886)

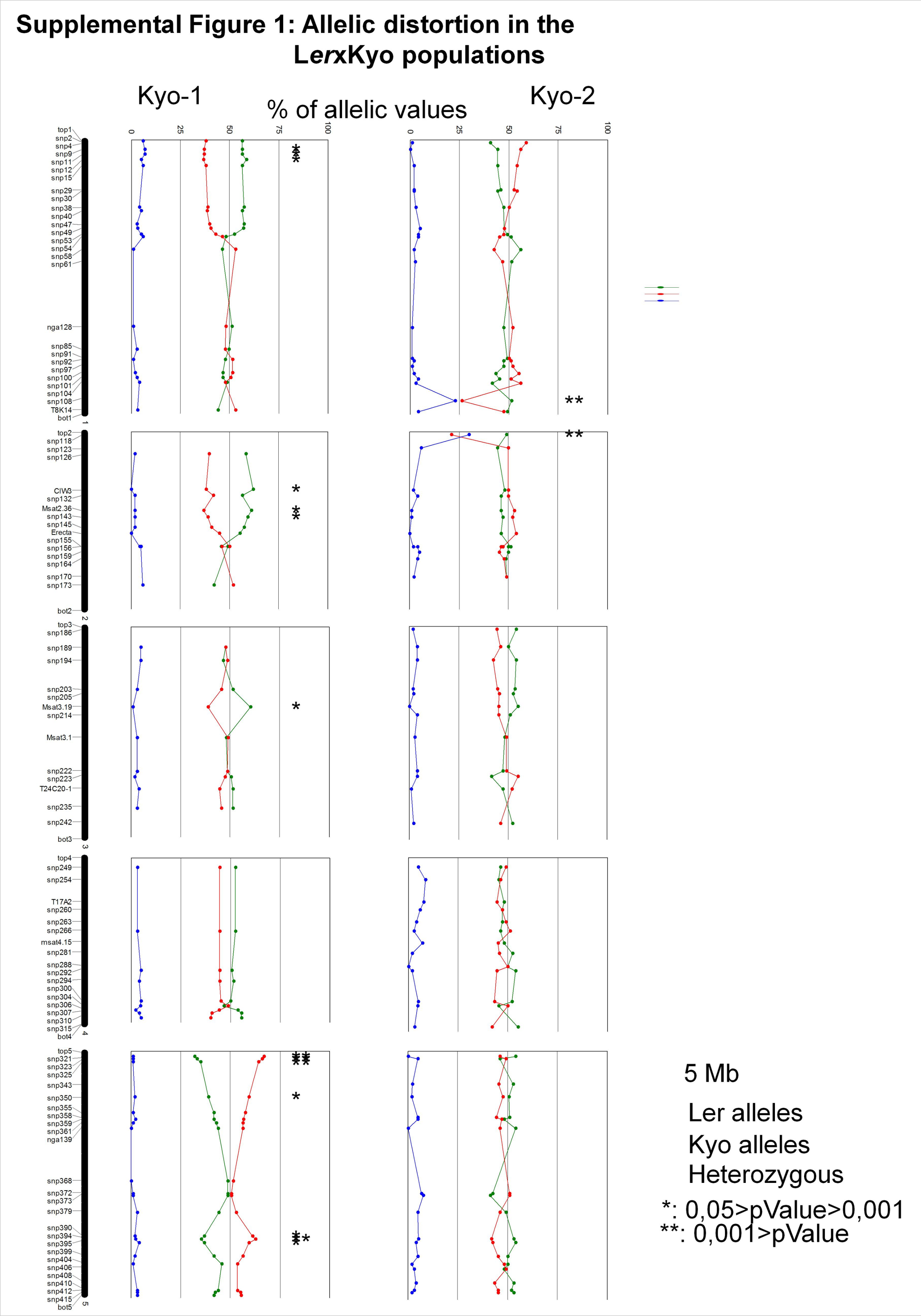

Supplement: Figure S1 — Segregation distortion represented by the frequency of each allele in the two mapping populations. * indicates where the deviation from 50% is significant. (TIF) [file pone.0020886.s001.tif]

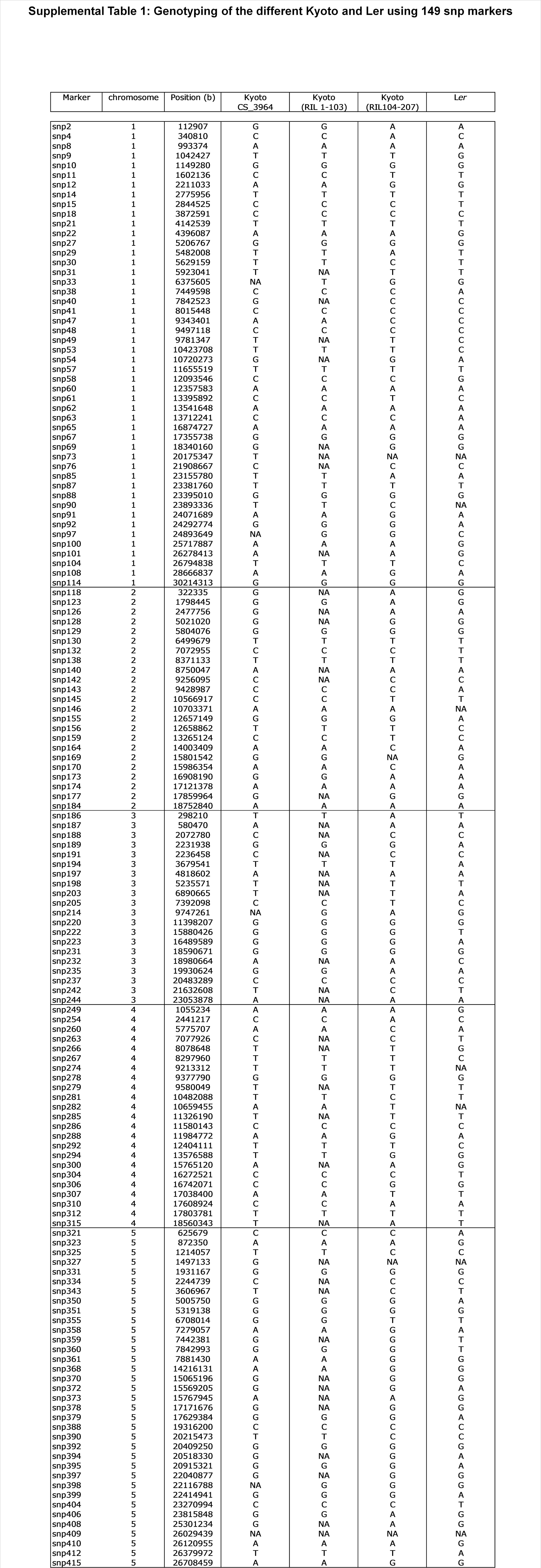

Supplement: Table S1 — Marker phenotypes of the parental lines. (TIF) [file pone.0020886.s002.tif]
